# Four decades applying clinical physiology to respiratory medicine: a personal account of occasional certainties and perennial doubts

**DOI:** 10.36416/1806-3756/e20260373

**Published:** 2026-09-14

**Authors:** José Alberto Neder

**Affiliations:** 1. Respiratory Investigation Unit, Kingston General Hospital, Division of Respirology, Department of Medicine, Queen’s University, Kingston (ON) Canada.; 2. Pulmonary Function Laboratory, Hotel Dieu Hospital, Kingston Health Sciences Center, Kingston (ON) Canada; 3. Pulmonary Rehabilitation Center, Providence Care Hospital, Kingston Health Sciences Center, Kingston (ON) Canada.

## INTRODUCTION

Respiratory physiology is arguably one of the most fascinating topics in Internal Medicine. Many young doctors attracted to the field of Pulmonology were at some point influenced by the blend of biology, physics, biochemistry, applied mathematics, and clinical acumen required to interpret pulmonary function and exercise tests cogently. Unfortunately, clinical respiratory physiology, once a dominant branch of knowledge,^1^ ^-^ ^3^ has been progressively overshadowed by the formidable advances in molecular biology, the omics revolution, and medical imaging. Nevertheless, the daily practice of Respiratory Medicine remains strongly anchored in information derived from the pulmonary function testing (PFT) laboratory; clinical decision-making still relies largely on the pathophysiological underpinnings of lung diseases. Despite its prominent role in driving clinical choices, less and less applied physiology is taught today; modern pulmonologists often grasp only superficial and frequently erroneous concepts. In fact, it is not uncommon to find residents who are convinced that they already master PFT interpretation even before starting their specialty training nowadays! The pervading influence of physiologists with a clinical bent, rather than clinicians with a physiological interest, and epidemiologists with little clinical insight has further muddied the waters.

Over several decades across three continents, I had the opportunity to systematically record the “post-PFT reading outcome” in multiple diagnostic and prognostic contexts, i.e., the extent to which physiological insights made clinical sense and positively impacted clinical decision-making. Perhaps even more important was the sobering recognition that, in many circumstances, my physiological impressions were imprecise, or overtly wrong, sometimes leading to mistakes and prompting further futile testing. The intent of this concise opinion manuscript, therefore, is to provide a factual account of my little *À la recherche du temps perdu* as I am leaving the building of clinical respiratory (patho)physiology in the closing stages of my career. I will focus on the key clinical decision nodes in Respiratory Medicine most affected by PFT/exercise testing results. For space reasons, I will skip other scenarios in which clinical physiology is invariably helpful, such as sleep medicine, intensive care, and non-invasive ventilation, to name only a few. For the experts in the field, I plead guilty to oversimplifications and to blending experimental and empirical evidence. At the bare minimum, I hope the final product proves useful to future generations of pulmonologists in making fewer mistakes than I did when translating respiratory functional assessment into daily clinical practice.^4^

## FRAMING THE MINDSET TO PROPERLY TRANSLATE PHYSIOLOGY TO CLINIC

A *sine qua non* is the tacit recognition that despite the maxim that “physiology never lies”, it can be quite shortsighted in the absence of external clues, i.e., pre-test clinical information. Physiological assessments more frequently describe patterns rather than point to a specific diagnosis (though there are honorable exceptions!). This should not prompt nihilism or open an excessively long list of differentials when interpreting testing results; for example, if all potential causes of a low transfer factor (DL_CO_) are cited in a report,^5^ it might ignite medically related anxiety and trigger multiple costly investigations. Use common sense; emphasize the most obvious: for many smokers, once anemia and recent smoking are ruled out, emphysema burden-rather than pulmonary vascular disease, consolidation, or a right-to-left shunt-should be prioritized. 

Be very cognizant of the multiple different resting and exercise patterns that may arise from a given disease or a combination of diseases, and try your best to weigh their potential individual contributions to the final picture; e.g., a seemingly “innocent” pattern of preserved dynamic and static lung volumes may coexist with profound impairment in gas exchange efficiency in patients with combined pulmonary fibrosis and emphysema; restriction may arise from heart failure rather than from trivial parenchymal reticulation in mild interstitial lung disease (ILD); and excessive ventilation during exercise in a patient with pulmonary vascular disease might be blunted by mechanical constraints brought be coexistent chronic obstructive pulmonary disease (COPD). The message is to always exercise humility, perhaps with a touch of educated guess, as we usually do when we do not have all the pieces of the puzzle.

## THE TESTING READER AND THE REQUESTER’S PERSPECTIVES

From the above, it is crystal clear that fewer errors are made when test interpretation is informed by clinical information; although this sounds like a cliché, it is simply paramount in interpreting most “advanced” PFTs and virtually any cardiopulmonary exercise test (CPET). If you are the sender (the reader) and little/no pre-test information is available, be sure to explain to the recipient (the requester) why your report is less informative and specific than it could ideally be. 

As a reader, make every effort to extract as much information as possible before reading the test, for example, by designing a test requisition that “squeezes” the requester to provide a bit of extra information or by accessing electronic medical records, amongst other strategies. I learned a lot, hopefully decreasing my mistakes, by linking function to structure; if feasible, review all recent imaging available. Use plain language and aim to answer the underlying clinical question; do not be dogmatic, avoiding cryptic elaborations to impress the recipient with your physiological knowledge. 

As a requester, you should be aware that, unlike many biochemical/blood tests, translating lung function and CPET results to an individual patient might not be as straightforward as desired; in many circumstances, the actual meaning of the test results would only emerge after your cognitive mediation. In a snapshot: the reader’s struggle is to grasp the whole from a part, whereas the requester’s battle is to understand the part knowing the whole.

## ESTABLISHING NORMALCY AND GRADING THE ABNORMAL: THE VARIEGATED SHADES OF GRAY

Here come two unwelcome guests to the subject: mathematics and statistics. Perhaps a good example is the FEV_1_/FVC conundrum in defining airflow obstruction: neither 0.7 (in adults) nor the statistical lower limit of normal (LLN)^6^ will always yield the unquestionable verdict on whether obstruction is present.^7^ Many smokers seen by pulmonologists who are in the so-called “gray zone” (0.7 < FEV_1_/FVC ≥ LLN) will have clear clinical, anatomical, and other functional evidence of COPD, but a good fraction will not.^8^ Using “slow” VC instead of FVC is double-edged: it may unravel obstruction in young and obese subjects but may grossly overdiagnose obstruction in the elderly.^9^ To further complicate things, smoking-related airway and alveolar disease may occur without obstruction; the preserved ratio impaired spirometry (PRISm) pattern^10^ and the patient with dyspnea and preserved FEV_1_ but with low DL_CO_ ^11^ highlight the limitations of focusing only on the FEV_1_/FVC ratio.^12^

Regardless of the metric, a borderline positive or negative test result around a numerical threshold should be valued depending on clinical input; for example, a DL_CO_ just slightly above the LLN in a young subject with suspected pulmonary embolism, or a trend to high lung volumes in a subject who has gone allogeneic stem cell transplant for leukemia (incipient bronchiolitis obliterans syndrome?). The advent of “global” lung function prediction values and the emphasis on race-neutral equations remain subjects of controversy; unrestricted adoption of these values may increase the frequency of subjects who show a non-specific pattern and/or mild restriction. Grades of lung function impairment based on z-scores^6^ have been benchmarked against mortality,^13^ not morbidity (e.g., exertional dyspnea severity); in practice, the intervals used to define mild, moderate, and severe impairment are too wide, reducing granularity compared with older schemes based on percentage of predicted value. My advice is to avoid strong statements about presence/absence of disease or, even worse, disease severity based solely on PFTs; be extra careful in trusting the cold, hard numbers in subjects at the extremes of age^14^ and height.

## MAKING FEWER MISTAKES IN THE LONGITUDINAL ASSESSMENT OF LUNG FUNCTION

This is another topic full of inconvenient truths: although some general recommendations can be made,^15^ in practice, this has been a field in which I have particularly struggled to make ends meet. More frequently than desired, PFTs “disagree” among themselves when providing a clear picture of whether the subject has improved, stabilized, or worsened. 

It is known that traditional spirometric indices (FEV_1_ and FVC) are more reproducible and less variable over time than “static” lung volumes by body plethysmography. However, these tests might be useful in untangling the meaning of a change in FVC: an increase in the “ceiling” (higher TLC) or a decrease in the “floor” (lower RV) has different implications in a patient with a mixed obstructive-restrictive defect. 

DL_CO_ is also seen more positively as a follow-up test, but when changes in alveolar volume (Va), whether physiological or artifactual, are not properly taken into consideration, mistakes will certainly be made.^16^ The effects of variations in body weight/obesity severity might be unexpectedly large; a patient with stable ILD may look worse or better if obesity progresses or improves, respectively.^17^

Overall, I strongly advise plotting longitudinal data over time, allowing for the ready detection of trends: watch the film; do not stare at the picture.

## CRITICALLY APPRAISING INTERPRETIVE ALGORITHMS

Out of many mistakes and challenges in teaching interpretive algorithms without justified critiques from attentive learners, I gradually developed an aversion to applying them to PFTs and exercise tests (despite having proposed a few myself!). Any down-to-earth, clinically proven scheme will require a very large footnote with exceptions to the rules. 

As a recent example, the 2022 European Respiratory Society/American Thoracic Society recommendations for PFT interpretation rely heavily on ratios (RV/TLC and functional residual capacity [FRC]/TLC) to distinguish hyperinflation from naturally large lung volumes when TLC is increased, and simple restriction from complex restriction when TLC is reduced.^13^ In practice, however, things are much more complicated; ratios are always dangerous, and in many circumstances, dissociations between these volumes represent different patterns of fibrosis deposition in ILD (central versus peripheral) or the effects of obesity and associated air trapping, to name only a few. This scheme also states that a normal FVC virtually excludes restriction; this is simply not true, as many patients with fairly extensive ILD show normal/near normal FVC due to a relatively low TLC coexisting with preserved or low RV/TLC. The document also fails to recognize that a proper interpretation of K_CO_ in those with low Va requires the latter to be related to TLC (see the section “The forgotten, sometimes very useful, PFTs”).^15^

An inherent issue with strict flowcharts is that they cannot account for multiple, often competing abnormalities that produce a picture that does not fit perfectly with the expected profile. Moreover, one should not underestimate the large confounding effects of the obesity pandemic in a progressively elderly population.^14^ The bottom line is the relevance of maintaining a critical eye when applying interpretation “guidelines”; if you depart widely from conventional recommendations, however, be sure to justify it properly. 

## VALUING THE ODD

Those interpreting many PFTs in daily practice might soon succumb to the “airplane pilot effect”: after dozens of tedious tasks, a nuanced situation that requires careful attention might be grossly overlooked, leading to mistakes. I invariably learned from the unexpected, e.g., complex restriction in a smoker with known COPD, the pattern of extraparenchymal restriction in a patient with ILD, a markedly reduced inspiratory capacity associated with high FRC/TLC in a never-smoker, or a blunted ventilatory response to exertion in a patient with pulmonary vascular disease, to name only a few. Once faced with the unexpected, do not fight with the numbers; instead, look for alternative explanations, while remaining open to the possibility that we may still not have come up with a reasonable explanation for some unusual findings. Strange and convoluted cases are invaluable windows through which to learn something new that, later on, might differentiate you from the crowd. 

## CPET IN RESPIRATORY MEDICINE: CLINICALLY USEFUL, BUT GROSSLY OVERSOLD

Dyspnea on exertion, usually multifactorial, is the commonest cause for a referral to a “lung” doctor.^18^ Thus, there is strong appeal for a test that claims to assess the integrated cardiopulmonary responses to exercise. Several decades devoted to (non- or minimally invasive) CPET taught me to apply the test with extra care in subjects with multiple potential causes of dyspnea and/or exercise intolerance.^19^

As a rule of thumb, CPET is more useful for ruling out major cardiorespiratory disease than for unequivocally pointing to a syndromic diagnosis-unless you are prepared to interpret clusters of abnormalities in light of all available clinical information.^20^ I wrote detailed accounts of CPET’s limitations and pitfalls in Respiratory Medicine^21^ ^,^ ^22^; as expected, they were not particularly popular amongst those profiting intellectually or materially from the status quo…It suffices to say that, since its inception, there has been a fundamental issue with the test’s conceptual approach: CPET was developed with a strong physiological, rather than clinical, eye. The major consequence was the belief that the task was to discover the system limiting maximal exercise capacity, rather than understanding why the respiratory patient reports exertional dyspnea, as is the case for most patients seen by pulmonologists.^23^ We have recently made some attempts to redirect the test towards a truly mechanistic framework focused on unveiling the deep roots of breathlessness^24^ ^-^ ^26^; whether this change in paradigms will overcome entrenched beliefs and vested interests remains to be seen.

## THE FORGOTTEN, SOMETIMES VERY USEFUL, PFTS

There are a few “old-fashioned” tests, usually available only in specialized centers, that might prove quite useful in challenging cases. Quantifying the wasted ventilation in the physiological dead space is a good example, since a high dead space/tidal volume ratio is a major cause of exertional dyspnea^18^ ^,^ ^27^; this information cannot be obtained with any other test. 

I had mixed experiences with shunt estimation using 100% O_2_; although borderline or slightly elevated values are difficult to interpret, a normal test can be useful in investigating unexplained hypoxemia. Comparing gas transfer measured by CO and nitric oxide might shed light on the relevance of the vascular component.^28^ Looking at the difference between Va (provided an adequate inspiratory volume) and TLC by body plethysmography yields an estimate of the poorly communicating fraction, an indirect index of ventilation-distribution abnormalities across peripheral airways.^29^ This might be helpful in detecting airway disease (in addition to interpreting K_CO_ properly) when their consequences are blurred by coexistent restriction, for example. 

Sniff inspiratory pressure is more sensitive and specific than maximal inspiratory pressure (MIP) in detecting diaphragm weakness; MIP is strongly influenced by the activation of accessory inspiratory muscles. This non-exhaustive list illustrates the importance of maintaining our capacity to deeply explore the functional properties of the respiratory system in well-resourced centers of excellence; thus, it is paramount to attract young clinicians and researchers to physiology before they are lost to more popular and profitable subspecialties.

## A (PARTIALLY) OPTIMISTIC CONCLUSION

All in all, it seems unlikely that clinical physiology will eventually become totally obsolete for those caring for cardiorespiratory patients; there is some well-founded hope that it will take a long time before traditional PFTs are finally considered part of the past. 

In addition to novel functional indexes, future generations will probably be exposed to new tests, such as those addressing the intricacies of pulmonary gas exchange, ventilation and perfusion distribution and their matching, and localized lung function tests; combining physiology with advanced functional respiratory imaging is an obvious avenue for obtaining unique information on structure-function relationships.^30^

Despite current excitement, any advances brought by artificial intelligence algorithms depend on our ability to train them using solid concepts rooted in knowledge already created, most of it dating from the “golden age” of physiology in the mid-to-late 20th century. Most importantly, however, the key relevance of medical input in determining a pragmatic pre-test likelihood of disease is unlikely to change much with time: clinical interpretation is fated to remain a N=1 study as long as we recognize the multifaceted complexities of the respiratory diseases.
